# Pulmonary Phthisis

**Published:** 1890-11-29

**Authors:** 


### THE PRACTITIONER'S MIRROR AND RETROSPECT.

[*The Editor will be glad to receive offers of co-operation and contributions from members of the profession. There is no doubt that much valuable material full of interest and instruction is at present lost to science. This is largely so in the case of* (1) *the younger and less-known members of the hospital staff* (2) *those connected with cottage hospitals, and* (3) *the great body of medical practitioners not attached to any institution. The co-operation of all is cordially invited to enable the Editor to make* THE HOSPITAL *of the utmost possible use and value to the profession. Specialists willing to review books on their special subjects are invited to communicate this fact at once. All letters should be addressed to* THE EDITOR, THE LODGE, PORCHESTER SQUARE, LONDON, W.]

### THE POST-GRADUATE COURSE AT THE BROMPTON CONSUMPTION HOSPITAL.

### PULMONARY PHTHISIS.

Dr. Percy Kidd, in commencing his lecture, said that he proposed to devote a few minutes to the consideration of the pathological anatomy of pulmonary phthisis, especially in its relation to the diagnosis and prognosis of the disease. He would only dwell on the gross anatomy of the subject as far as it helped us to form an opinion on the diagnosis and prognosis of individual cases. Tuberculosis is a specific infectious cell growth, characterised by the tendency: on the one hand to the process of caseous degeneration or necrosis; and on the other hand to the formation of fibrous tissue. Caseation or necrosis is nearly always present at some period of the disease. The formation of fibrous tissue is less common, and is usually associated with some necrosis. Tubercle appears either in the form of small nodules at one or more centres, or occasionally shows itself as a diffuse infiltration. Frequently its infectious nature is shown by a gradual extentension from one or more centres. The pulmonary lesions consist of a nodular consolidation, which may be simple or compound, and in the latter case has a racemose appearance; this condition is due to the grouping of tubercles in relation to the branches of the bronchioles. In the immediate neighbourhood of the original tubercular foci, there is usually some bronchitis or catarrh of the lung. The first part of the lung to be affected is usually the apex, or an area just below the apex, the deposit very rarely, primarily, appearing at the base. When once a nodule is present in the centre of the lung tissue, similar ones spring up in the neighbourhood. The degree of consolidation and the amount of collapse of the surrounding tissues are very various. The progress of the deposit is usually as follows: First, a cellular growth, the outlines of the individual cells in the centre of this nodule disappear, the whole deposit then undergoing necrosis and becoming caseous. For a long time writers on the subject associated this degeneration with the obstruction of the blood vessels, there being usually no vessels to be seen in the growth. Now, however, the necrosis is generally said to be due to some toxic action of the tubercle bacilli, either direct or as the result of some chemical poison produced by them. The caseous mass thus formed may remain quiescent for some time, or it may be come surrounded by a fibro-cellular zone of tissue. In certain cases a deposit of lime salts may occur in it and the mass becomes a calcified patch; this represents arrest of the disease. More usually, however, the degenerated mass breaks down, and opening into a bronchial tube is discharged, a small cavity being thus produced. Several cavities being formed in this way tend to fuse together, as the result of which part of a lobe, or even the whole lung, may become excavated. Usually these vomicæ have bands of fibrous tissue stretching across their interior. Caseous degeneration may appear over a large area, producing a caseous pneumonia. This condition is possibly due to isolated nodular deposits of tubercle which have amalgamated with some surrounding patches of pneumonia. The peripheral zone of the tubercular nodule consists of bundles of fibrous tissue and spindle cells. When calcification occurs these form a dense fibrous capsule to the mass; if a vomica has been produced they form the wall of the cavity. As the result of this fibrous tissue formation, contractions of the surrounding lung may occur. Chronic cases of phthisis are usually associated with a considerable deposit of pigment, which is mainly the result of inhaled carbon particles, possibly together with some changed blood. Although the caseous and fibrotic changes have been described separately, they usually proceed together in the diseased regions. The secondary results of tubercular deposit in the pulmonary tissues are the following: pleurisy, emphysema, bronchial dilatation (bronchiectasis), aneurysm of the branches of the pulmonary artery. The pleurisy may be dry or with exudation, the former being the more common. As a rule, the serous covering of the apex of the lung is first affected, and as a result of this, adhesions occur which usually first are formed in the scapular regions. The pleurisy may be local or general, and it may either precede the development of phthisis or follow it. The pleural affection is usually set up by the direct extension of inflammation from the nodules in the lung to the surface, or by the direct transmission of the virus through the lymphatics. As the result of the pleuritic adhesions hyper-distension or emphysema of the parts may follow. This is especially marked in the front area of the lungs from the posterior parts being fixed by adhesions. As the result of the emphysema the lung becomes dry and pale, forming a less favourable soil for the further development of tubercle. Although this condition is favourable as far as the pulmonary disease is concerned, the obliteration of the small vessels in the emphysematous areas increases the work of the already over-burdened right heart. On the whole the emphysema may nevertheless be considered a more or less favourable sign. The bronchial dilatation usually occurs in very chronic cases of phthisis, and occasionally is the factor in the formation of tubercular cavities. The development of pulmonary aneurysms, especially with reference to hæmopytsis, is of great importance, but can only be mentioned in this place. With regard to the manner of extension of the disease through the lungs, in the above racemose type, the process seems to commence in the bronchioles. There is reason to believe that the several affected areas are due to the distribution of the bronchial tubes. What may be considered the areas of election of the disease in order of frequency are as follows: (1) the apex of the upper lobe, (2) the apex of the lower lobe, (3) the lower part of the upper lobe. The first one in this country to describe this was Dr. Ewart, and more recently a paper has been published by Dr. Fowler on the subject. The result of these investigations of the way the process extends in the lungs is a further proof that it is through the bronchial tubes; the lesions often corresponding to the distribution of these passages in the lung. In early stages in many cases circumscribed lesions of the upper and lower lobes are found, with a considerable amount of normal pulmonary tissue between. This condition removes the idea of a direct extension of the process from one to neighbouring areas. This process of secondary infection has been described by Dr. Reginald Thompson as an "expression of the auto-infection of the lungs through the bronchial tubes." The earliest physical signs may be slightly altered breathing at the apex of one lung, with possibly a diminished expansion of the chest walls. The presence of pulmonary consolidation at its earliest period may give no evidence of its existence. The changes of the respiratory sounds vary much with the amount of secretion in the tubes and the collapse of lung tissue present. A sub-crepitant râle may be the only abnormal sign found, and this possibly only after making the patient cough. After consolidation has progressed to any extent, signs of an apical pneumonia may be present. An early stage of the disease may give the following signs over the seat of the lesion: moderate dulness to percussion, increased vocal resonance, weakness of inspiration and slight prolongation of expiration, râles may, or may not be present. The consolidation of phthisis in an early stage is nearly always associated with surrounding bronchitis; pleuritic signs may also very early show themselves. As the necrotic changes proceed, signs of excavation appear, usually, together with some dulness on percussion from the consolidation of the tissues around the cavities. The "cracked pot sound" when present, shows that the vomica is superficial and that it is in direct communication with the bronchus. Dr. Mitchell Bruce has described what he calls "the indiarubber ball sound;" this is a sucking sound which may occasionally be heard after the patient coughs. For the convenience of description the disease pathologically may be divided into three stages, namely, consolidation, softening, and excavation. Clinically, however, these divisions are of no value, for by the time consolidation is found, softening is also in progress. In cases of what are spoken of as very early phthisis, bacilli are present in the sputum, and therefore there must be excavation already in process of formation. The real danger to the patient does not occur so much during the third, that is the stage of excavation, as during the first stage when consolidation is in progress. During the third stage the patient may improve. The stages are, therefore, artificial distinctions, and of little value. In all cases of phthisis it is of great importance to examine the apices of the lower lobes, that is to say those regions of the lungs corresponding to the infra scapular fossæ. It is, however, very hard to diagnose cavities here on account of the proximity of the large bronchus going to the lower lobe. Frequently all the signs of a vomica which are present about the angle of a scapula, may be due to a localised patch of pneumonia in relation to the bronchus at this spot, and not to a cavity. Clinically, phthisis may be divided into the following groups or types: (1) The ordinary type, which may be described as a localised apical catarrh, followed by consolidation and a gradual extension of the disease downwards until more or less the whole of the upper lobe becomes affected. In an early stage corresponding morbid signs are found at the apex of the lower lobe. (2) The pneumonic type. This begins in a more or less acute way, with symptoms of pneumonia, especially of the left upper lobe. This is, however, not a very common type. (3) The broncho-pneumonic type. This begins in a more or less acute onset, with the presence of multiple tubercular nodules, which rapidly caseate and undergo softening. In this form the physical signs are very various and scattered, in some parts with tubular breathing and in others vesicular. This is usually accompanied with high pyrexia, profuse sweats, and rapid emaciation; in fact, the "galloping consumption" of older writers. All of these three types may develop into what we may consider the chronic type. (4) The chronic, fibroid, or contractile type is characterised by its very slow tendency to spread to the opposite lung. This form may be accompanied by a good deal of emphysema. As the result of the contraction of the diseased parts of the lung there may be considerable displacement of the heart. The diaphragm, and with it some of the abdominal viscera, may be drawn up into the thoracic cavity. (5) The emphysematous type. This is of importance on account of the difficulty of diagnosis. In these cases the apices of the lungs posteriorly should be carefully examined. There may possibly be slight dulness in these regions, together with general emphysematous signs. The presence of emphysema in young people with the history of hæmoptysis should be very suggestive of chronic phthisis. (6) The pleuritic type. In this variety the pleuritic signs may be the only ones obtainable. These may supervene on a pre-existing tubercular lesion, or the disease of the lung may be only recognised after subsidence of the fluid. The dry form is more frequent than the fluid one. The presence of pleurisy of both sides is strongly suggestive of tubercle, and with a history of hæmoptysis it is very important to examine the sputum for bacilli. (7) Basic phthisis, that is, when the discovered signs are limited to the bases of one or both lungs. Priminary basic phthisis is exceedingly rare, occuring once in about 400 or 500 cases. On this question many fallacies exist, as the signs which are only present at the base may be, and frequently are, secondary to old apical lesions which on account of the surrounding emphysema may not be diagnosed. Prognosis from the pathological and anatomical point is not of so much value as from the general condition of the patient. The predominance of fibrotic changes are good signs, especially when associated with contraction. Frequently well-defined dulness and contraction, as also the presence of emphysema about the morbid areas, point to a chronic process. So also pleuritic effusion may for a time arrest the progress of the disease. Extensive pleuritic adhesions are unfavourable, as they limit the movements of respiration. In conclusion, it must be noted that an apparently acute type may occasionally suddenly become chronic, and a chronic one equally rapidly become acute.

